# AMPK-autophagy inhibition sensitizes icaritin-induced anti-colorectal cancer cell activity

**DOI:** 10.18632/oncotarget.14718

**Published:** 2017-01-18

**Authors:** Chunxian Zhou, Jun Gu, Gang Zhang, Da Dong, Qunying Yang, Min-Bin Chen, Dongfeng Xu

**Affiliations:** ^1^ Department of Interventional Radiology, Wujiang Hospital Affiliated to Nantong University, Wujiang, Suzhou, China; ^2^ The Department of Orthopedics, The Second Affiliated Hospital of Soochow University, Suzhou, China; ^3^ Department of Oncology, Kunshan First People's Hospital Affiliated to Jiangsu University, Kunshan, 215300, China

**Keywords:** colorectal cancer, icaritin, autophagy, AMPK, chemosensitization

## Abstract

The current research studied the potential effect of autophagy on icaritin-induced anti-colorectal cancer (CRC) cell activity. Treatment of icaritin in both primary and established (HT-29) CRC cells induced feedback activation of autophagy, evidenced by p62 degradation, Beclin-1 and autophagy-related gene-5 (ATG-5) upregulation, as well as light chain 3B (LC3B)-GFP puncta formation. Pharmacological inhibiting of autophagy dramatically potentiated icaritin-induced CRC cell death and apoptosis. Meanwhile, shRNA-mediated knockdown of Beclin-1 or ATG-5 also sensitized icaritin-induced CRC cell death and apoptosis. Icaritin activated AMP-activated protein kinase (AMPK) signaling in CRC cells, functioning as the upstream signaling for autophagy activation. shRNA/siRNA-mediated knockdown of AMPKα1inhibited icaritin-induced autophagy activation, but exacerbated CRC cell death. On the other hand, the AMPK activator compound 13 (C13) or the autophagy activator MHY1485 attenuated icaritin-induced cytotoxicity. In nude mice, icaritin (oral administration)-induced HT-29 tumor growth inhibition was potentiated when combined with AMPKα1 shRNA knockdown in tumors. We conclude that feedback activation of AMPK-autophagy pathway could be a primary resistance factor of icaritin.

## INTRODUCTION

The colorectal cancer (CRC) has long been a major health problem in both Eastern and Western countries [1–3]. Hence, our group [10] and others [1, 8] have been focusing on exploring novel anti-CRC agents. Icaritin, the hydrolytic product of Icariin, is the main active ingredient of *Epimedium*, which is a known Traditional Chinese Medicine [11]. Studies have tested its biological functions in different experimental settings, including its anti-cancer activity [12–14]. For instance, icaritin was shown to inhibit proliferation of prostate cancer cells [12], breast cancer cells [13], as well as hepatocellular carcinoma (HCC) cells [15] and endometrial cancer cells [16]. Our recent study has demonstrated that icaritin could also inhibit CRC cells *in vitro* and *in vivo* [10]. At the molecular level, we found that icaritin activated JNK-dependent mitochondrial permeability transition pore (mPTP) necrosis pathway to kill CRC cells [10].

One important aim of the current study is to identify possible icaritin's resistance factor. We here focused on the potential involvement of autophagy in the process. Existing studies have displayed feedback activation of autophagy in many cancer cells following treatment of a variety of anti-cancer drugs [17–21], which could be a key resistance factor to inhibit cancer cell death and apoptosis [17, 21, 22]. Reversely, genetic or pharmacological inactivation of autophagy could then sensitize the anti-cancer activity by these anti-cancer drugs [17–22]. In the current study, we showed that autophagy inhibition dramatically sensitizes icaritin-induced anti-CRC cell activity.

## RESULTS

### Icaritin activates autophagy in human CRC cells

**Figure 1:**
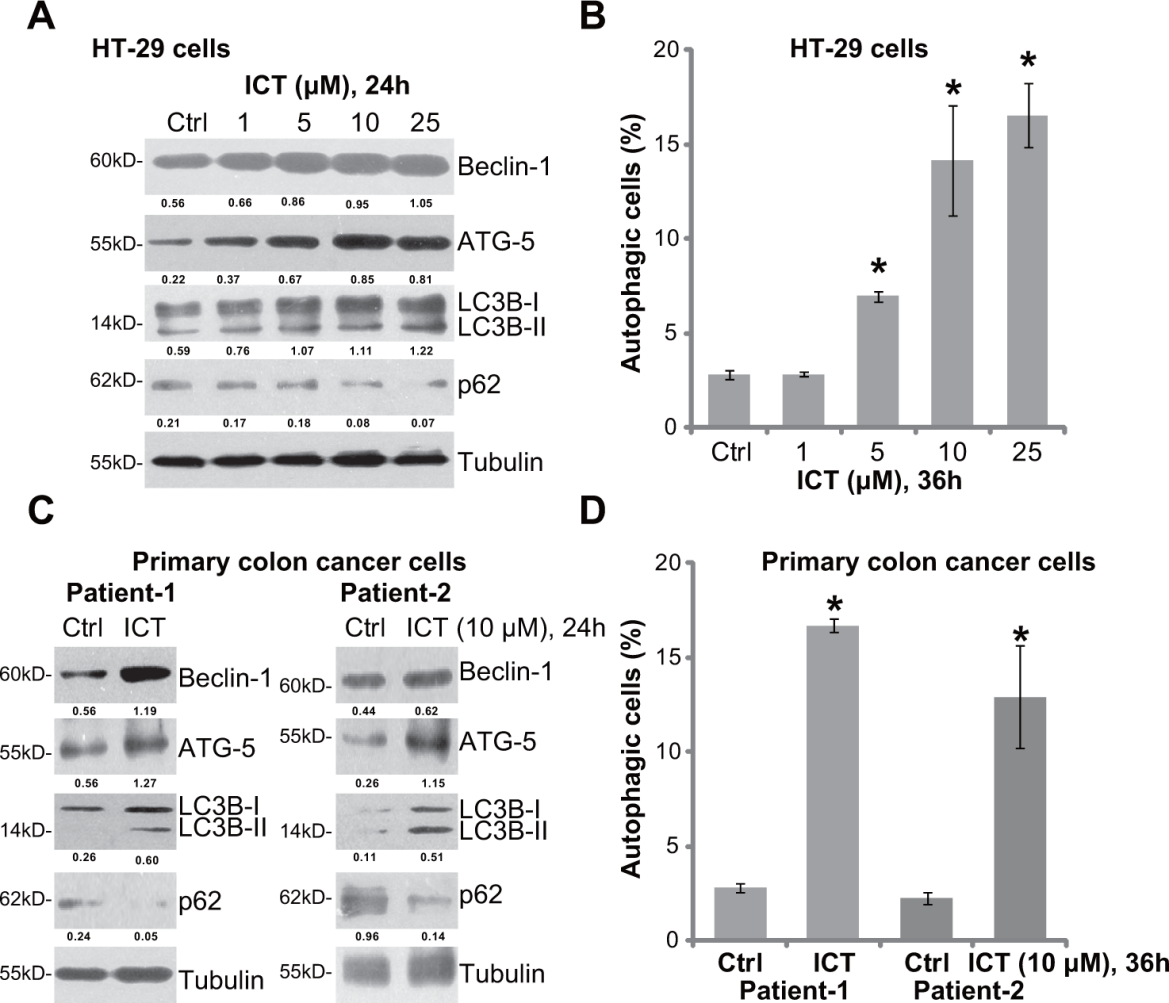
Icaritin activates autophagy in human CRC cells HT-29 cells (**A** and **B**) or the primary colon cancer cells (two lines, “Patient-1/−2”) (**C** and **D**), were either left untreated (“Ctrl”, same for all figures), or treated with applied concentration of icaritin (ICT, same for all figures) for indicated time; Expression of listed proteins was shown (A and C); Percentage of LC3B-GFP puncta positive cells, or autophagic cells, was also recorded (B and D). Expression of listed proteins was quantified and normalized to Tubulin (A and C). Data were expressed as mean ± standard deviation (SD), experiments were repeated five times. *n* = 5 for each assay. *p < 0.05 vs. “Ctrl” group.

In order to test the potential effect of icaritin on autophagy, Western blot assay was performed to test expression of autophagy-associated proteins in icaritin-treated cells. As demonstrated, treatment of icaritin in HT-29 cells dose-dependently upregulated Beclin-1, autophagy-related gene-5 (ATG-5) and light chain 3B-II (LC3B-II), but downregulated p62 (Figure 1A). Meanwhile, the percentage of LC3B-GFP puncta positive cells, or autophagic cells, was also significantly increased following icaritin (5–25 μM) treatment (Figure 1B). These results suggested autophagy activation in HT-29 cells after icaritin treatment [23–26]. Similarly, in two lines of primary colon cancer cells (patient-derived), icaritin (10 μM) treatment induced Beclin-1, ATG-5 and LC3B-II upregulation but p62 degradation (Figure 1C). Further, the number of autophagic cells was also significantly increased after icaritin treatment in the primary colon cancer cells (Figure 1D). Thus, these results indicate that icaritin induces autophagy activation in established and primary CRC cells.

### Autophagy inhibitors potentate icaritin-induced CRC cell death and apoptosis

**Figure 2:**
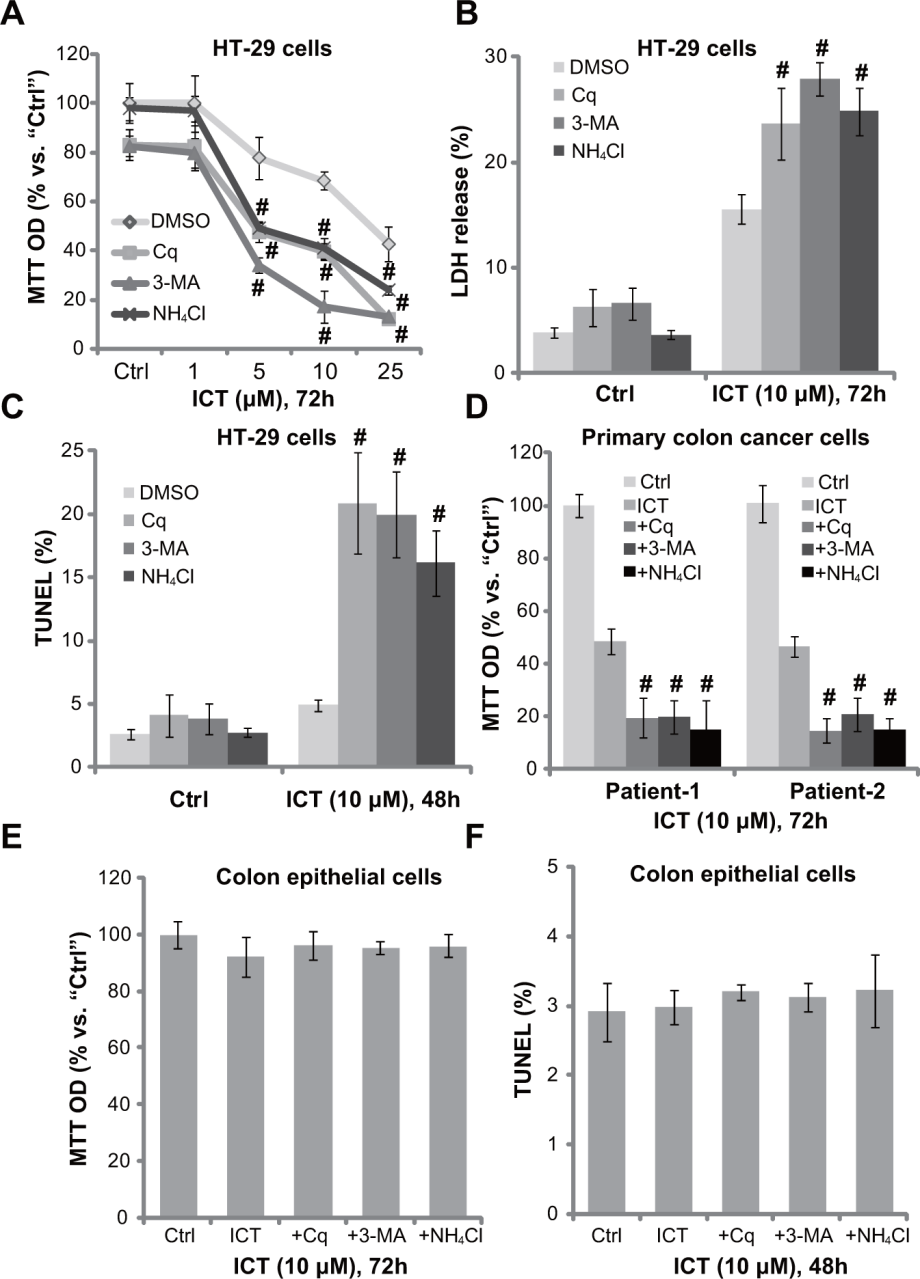
Autophagy inhibitors potentate icaritin-induced CRC cell death and apoptosis HT-29 cells (**A**–**C**), primary colon cancer cells (two lines, “Patient-1/−2”) (**D**), or the primary colon epithelial cells (**E** and **F**), pre-treated for 30 min with applied autophagy inhibitors: 3-methyladenine (3-MA, 1.0 mM), chloroquine (Cq, 5 μM) or ammonium chloride (NH_4_Cl, 2.5 mM), were subsequently treated with ICT (10 μM) for designated time; Cell viability, cell death and apoptosis were tested by MTT assay (A, D and E), LDH release assay (B) and TUNEL staining assay (C and F), respectively. Experiments in this figure were repeated three times, and similar results were obtained. “DMSO” stands for 0.1% DMSO of vehicle control. Data were expressed as mean ± SD, experiments were repeated five times. *n* = 5 for each assay. ^#^*p* < 0.05 vs. “DMSO” group.

To study the potential effect of autophagy in icaritin-mediated anti-CRC cell activity, various autophagy inhibitors were applied, including chloroquine (Cq), ammonium chloride (NH_4_Cl) and 3-methyaldenine (3-MA). MTT assay results showed that, in the presence of these autophagy inhibitors, icaritin-induced viability reduction was significantly potentiated (Figure 2A). The icaritin's IC50, the concentration that inhibits 50% of cell viability, decreased from over 20 μM to less than 5 μM with co-treatment of the autophagy inhibitors (Figure 2A). icaritin-induced HT-29 cell death, tested by the lactate dehydrogenase (LDH) release, was also significantly augmented with the autophagy inhibitors (Figure 2B). In line with our previous findings [10], treatment with icaritin (10 μM) alone failed to induce significant apoptosis activation in HT-29 cells (Figure 2C). Remarkably, when combined with the autophagy inhibitors, icaritin provoked dramatic apoptosis (Figure 2C), which was tested by the TUNEL staining assay (Figure 2C). In the primary colon cancer cells, the above autophagy inhibitors similarly potentiated icaritin-induced cell viability reduction (Figure 2D). Thus, pharmacological inhibition of autophagy potentates icaritin's cytotoxicity in CRC cells. On the other hand, in the primary human colon epithelial cells, treatment with icaritin or together with these autophagy inhibitors failed to induce significant cell viability reduction (Figure 2E) and apoptosis (Figure 2F). Thus, icaritin combination with the autophagy inhibitor was only cytotoxic to cancerous cells.

### Beclin-1 or ATG-5 shRNA knockdown sensitizes icaritin-induced cytotoxicity against CRC cells

**Figure 3:**
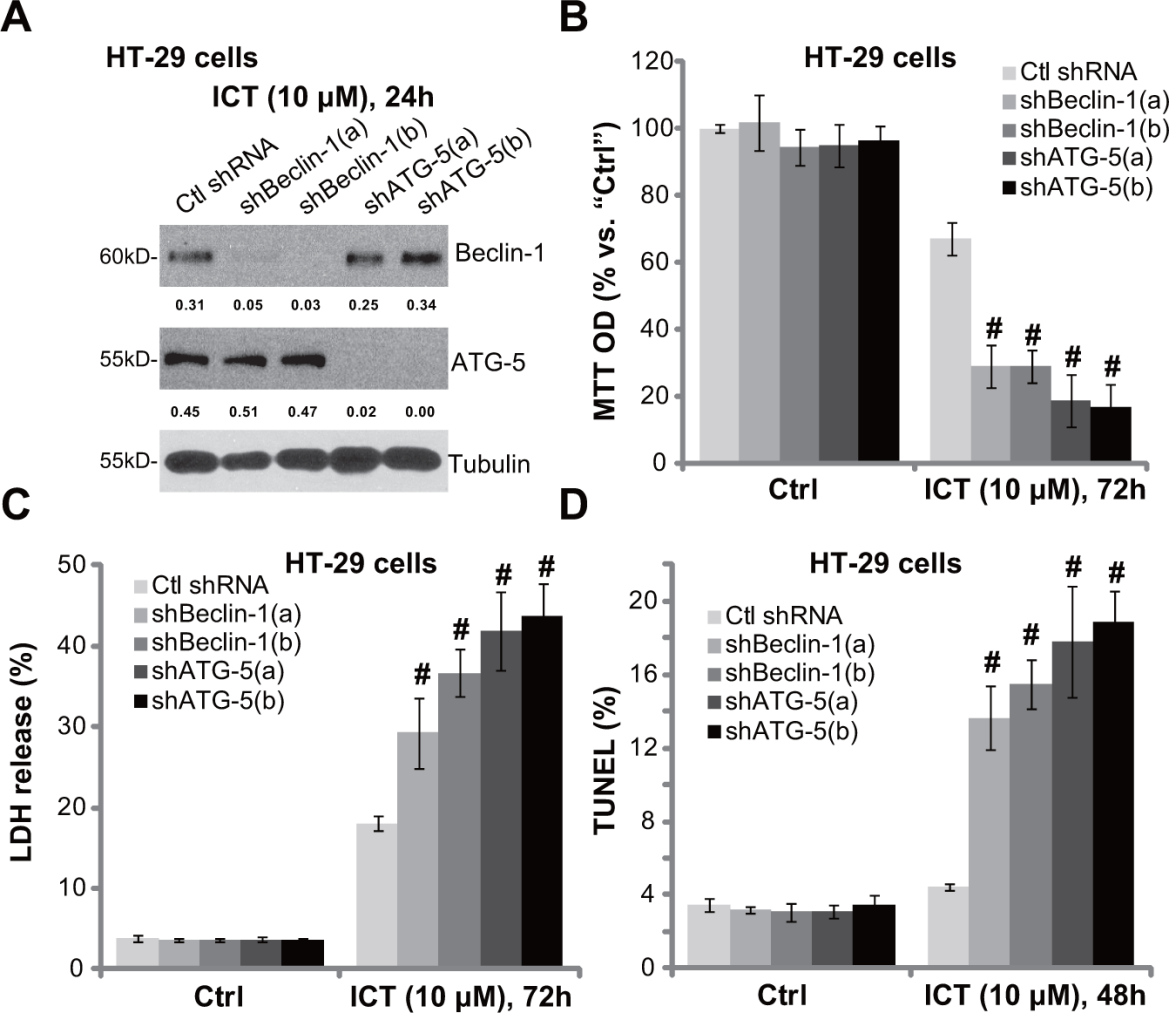
Beclin-1 or ATG-5 shRNA knockdown sensitizes icaritin-induced cytotoxicity against CRC cells The stably HT-29 cells with scramble non-sense control shRNA (“Ctl shRNA”), Beclin-1 shRNA (“shBeclin-1-a/b”) or ATG-5 shRNA (“sh ATG-5-a/b”) were treated with ICT (10 μM) for designated time; Expression of listed proteins was tested by Western blot assay (**A**); Cell viability (**B**), cell death (**C**) and apoptosis (**D**) were also tested. Expression of Beclin-1 and ATG-5 was quantified and normalized to Tubulin (A). Data were expressed as mean ± SD, experiments were repeated three times. *n* = 5 for each assay. ^#^*p* < 0.05 vs. “Ctl shRNA” group.

The above pharmacological evidences suggest that autophagy activation possibly serves as a potential resistance factor of icaritin. To exclude the possible off-target effect of the applied autophagy inhibitors, shRNA method was utilized to silence key autophagy-associated proteins, including Beclin-1 [27] and ATG-5 [28]. As demonstrated in Figure 3A, the two different Beclin-1 shRNAs (“a/b”) both efficiently and stably downregulated Beclin-1 in HT-29 cells. Similarly, the two specific shRNAs (“a/b”) [29] also silenced ATG-5 in HT-29 cells (Figure 3A). Remarkably, icaritin-induced HT-29 cell viability reduction (Figure 3B), cell death (Figure 3C) and apoptosis (Figure 3D) were significantly intensified with Beclin-1 or ATG-5 shRNA knockdown. Yet, knockdown of these proteins alone showed no significant effect on cell survival and apoptosis (Figure 3B and 3D). These genetic evidences further suggest that autophagy inhibition could sensitize icaritin's activity in CRC cells.

### Icaritin activates AMPK as upstream of autophagy in CRC cells

**Figure 4:**
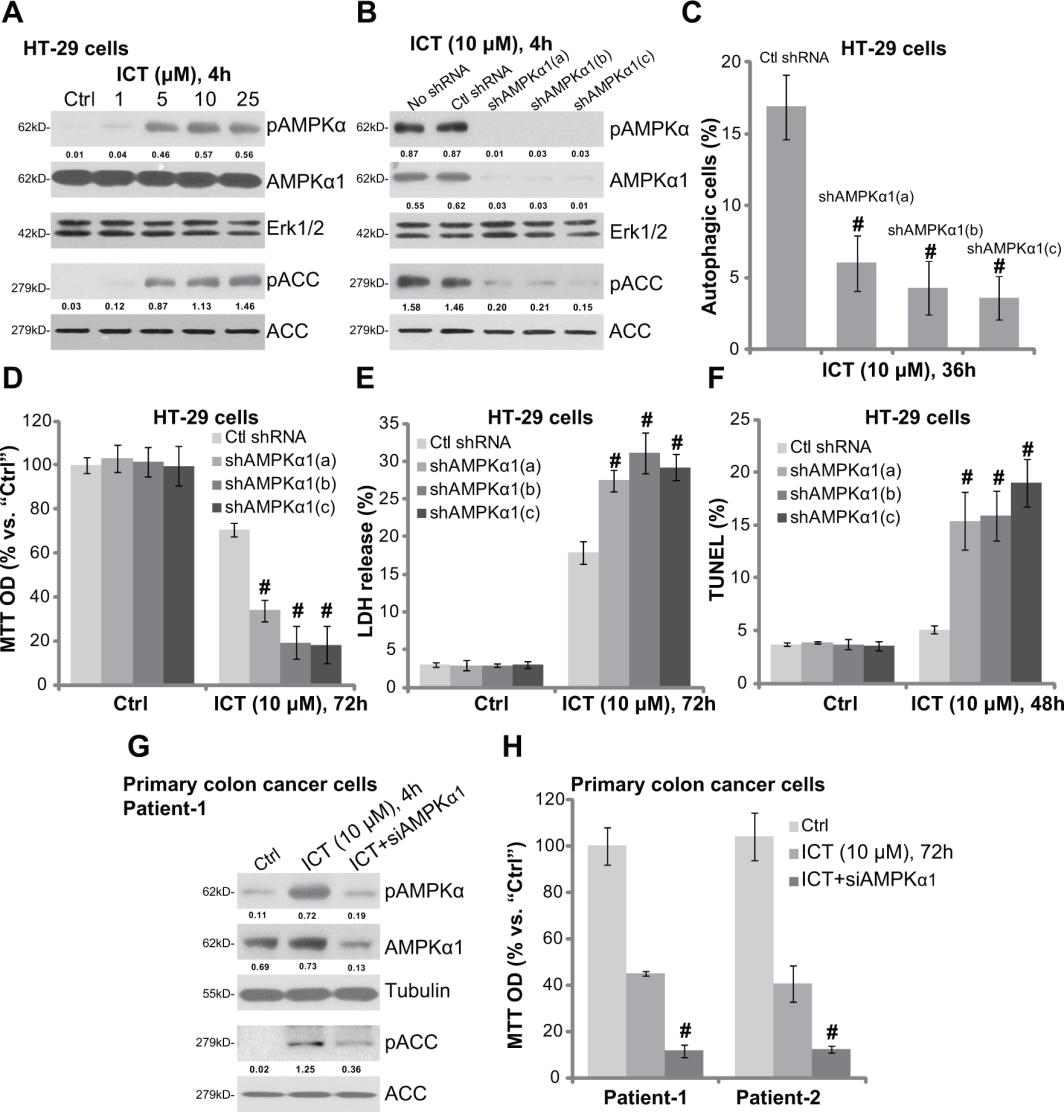
Icaritin activates AMPK as upstream of autophagy in CRC cells HT-29 cells were treated with applied concentration of ICT for 4 hours, expression of listed proteins was shown (**A**); The stable HT-29 cells, expressing scramble non-sense control shRNA (“Ctl shRNA”) or indicated AMPKα1 shRNA (“a/b/c”), were treated with/out ICT (10 μM) for designated time; Expression of listed proteins was shown (**B**); Percentage of LC3B-GFP puncta cells (“autophagic cells”) was recorded (**C**); Cell viability (**D**), cell death (**E**) and apoptosis (**F**) were also tested. Primary colon cancer cells with/out AMPKα1 siRNA (siAMPKα1, 200 nM, 48 hours) were treated with ICT (10 μM) for designated time; Expression of listed proteins was shown (**G**); Cell viability was also tested (**H**). Phosphorylation (“p”) of AMPKα (vs. loading Erk1/2 or Tubulin) and ACC (vs. regular ACC) was quantified. Data were expressed as mean ± SD, experiments were repeated three times. *n* = 5 for each assay. ^#^*p* < 0.05 vs. “ICT” only group.

Next, we explored the potential upstream signaling responsible for autophagy activation by icaritin. Growing evidences have suggested that AMP-activated protein kinase (AMPK) activation could provoke autophagy under a number of stimuli [30–32]. Here, we showed that icaritin treatment in HT-29 cells dose-dependently induced AMPK activation, which was tested by phosphorylation (“p”) of AMPKα (Thr-172) and its major downstream kinase ACC (acetyl-CoA carboxylase, Ser-79) (Figure 4A). Expression of regular AMPKα1 and ACC was unchanged following icaritin treatment (Figure 4A). To study the activity of AMPK in icaritin-induced autophagy activation, we again utilized shRNA method to stably knockdown AMPKα1 in HT-29 cells. As demonstrated, the three targeted-shRNAs (“a/b/c”) dramatically downregulated AMPKα1 in HT-29 cells (Figure 4B). Consequently, icaritin-induced AMPK activation, or AMPKα-ACC phosphorylation, was also dramatically inhibited (Figure 4B). Remarkably, AMPKα1 knockdown almost blocked icaritin-induced autophagy activation in HT-29 cells (Figure 4C), indicating that AMPK activation is required for icaritin-induced autophagy activation.

If AMPK activation is the upstream signaling for icaritin-mediated autophagy activation, blockage of AMPK should also potentate icaritin's cytotoxicity in CRC cells. Indeed, we showed that icaritin-induced viability reduction (Figure 4D), cell death (Figure 4E) and apoptosis (Figure 4F) were remarkably augmented in AMPKα1-shRNA-expressing HT-29 cells. In another word, AMPKα1 silence could significantly facilitate icaritin-induced lethality against HT-29 cells (Figure 4D–4F). In the primary colon cancer cells, siRNA was applied to temporary knockdown AMPKα1. The targeted siRNA decreased AMPKα1 expression and inhibited icaritin-induced AMPK activation (Figure 4G). Significantly, icaritin-induced cytotoxicity, or viability reduction, was also exacerbated with AMPKα1 siRNA knockdown in the primary cancer cells (Figure 4H). Collectively, these results indicate that icaritin-induced AMPK activation serves as the upstream signaling for subsequent autophagy activation. Reversely, AMPK inactivation by siRNA/shRNA knockdown of AMPKα1 significantly potentiates icaritin-induced cytotoxicity in CRC cells.

### AMPK-autophagy activators attenuate icaritin's cytotoxicity in CRC cells

**Figure 5:**
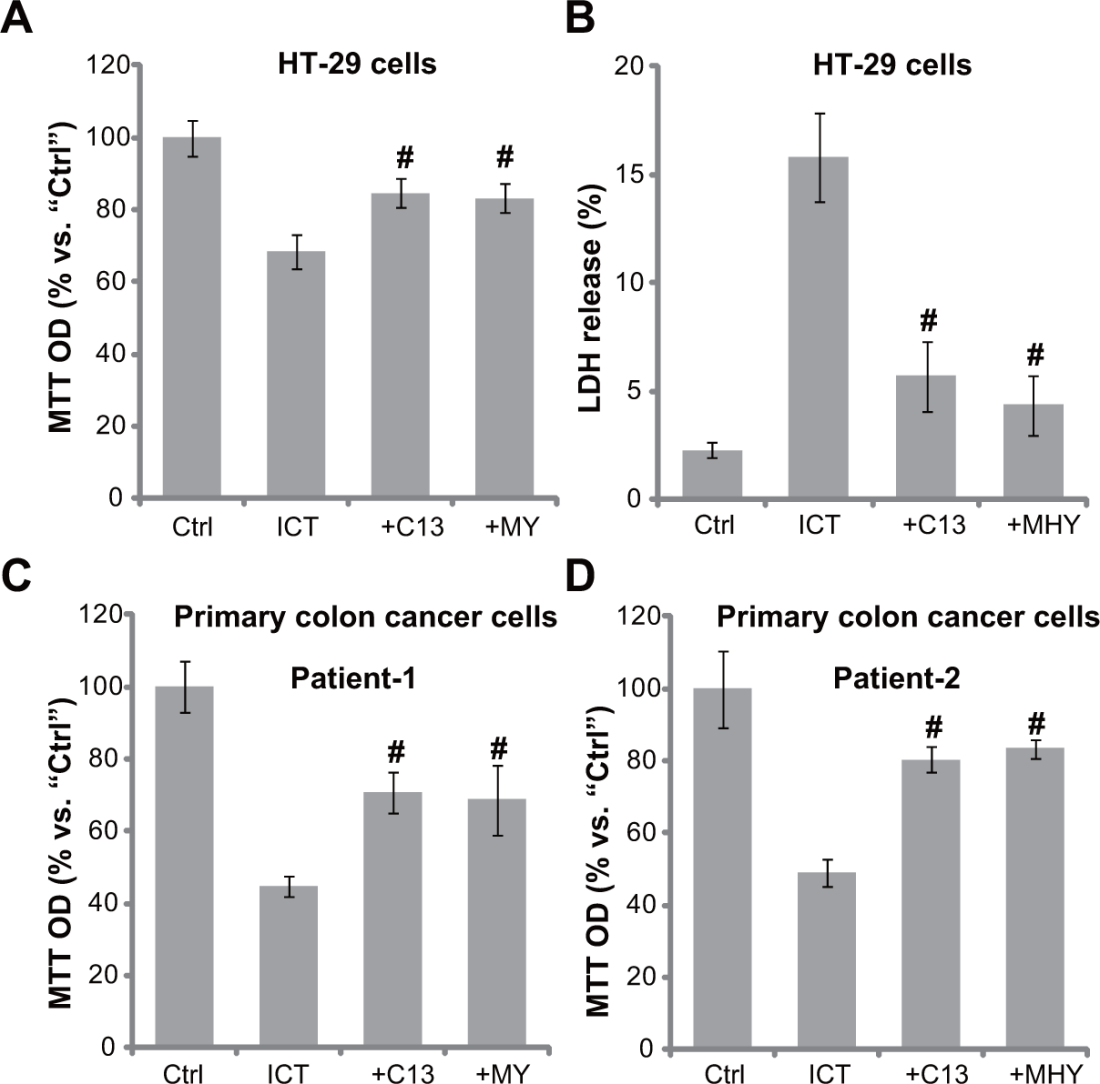
AMPK-autophagy activators attenuate icaritin's cytotoxicity in CRC cells HT-29 cells (**A**–**B**) or the primary colon cancer cells (two lines, “Patient-1/−2”) (**C** and **D**) were pre-treated for 30 min with the AMPK activator Compound 13 (“+C13”, 10 μM) or the autophagy activator MHY1485 (“+MHY”, 10 μM), followed by ICT (10 μM) treatment for 72 hours; Cell viability and cell death were tested by MTT assay (A, C and D) and LDH release assay (B), respectively. Data were expressed as mean ± SD, experiments were repeated five times. *n* = 5 for each assay. ^#^*p* < 0.05 vs. “ICT” only group.

Based on the results above, we would speculate that forced-activation of AMPK-autophagy pathway should attenuate icaritin's cytotoxicity. In the current study, an α1 specific AMPK activator Compound 13 (C13) [33, 34] and an autophagy activator MHY1485 [35] were utilized. Results showed that pre-treatment of the two activators indeed inhibited icaritin-induced HT-29 cell viability reduction (Figure 5A) and cell death (Figure 5B). Similarly in the primary colon cancer cells, Compound 13 and MHY1485 attenuated icaritin's lethality (Figure 5C and 5D). Therefore, pharmacologic forced-activation of AMPK-autophagy pathway attenuates icaritin's cytotoxicity in CRC cells.

### AMPKα1 silence sensitizes icaritin-induced anti-HT-29 tumor activity *in vivo*

**Figure 6:**
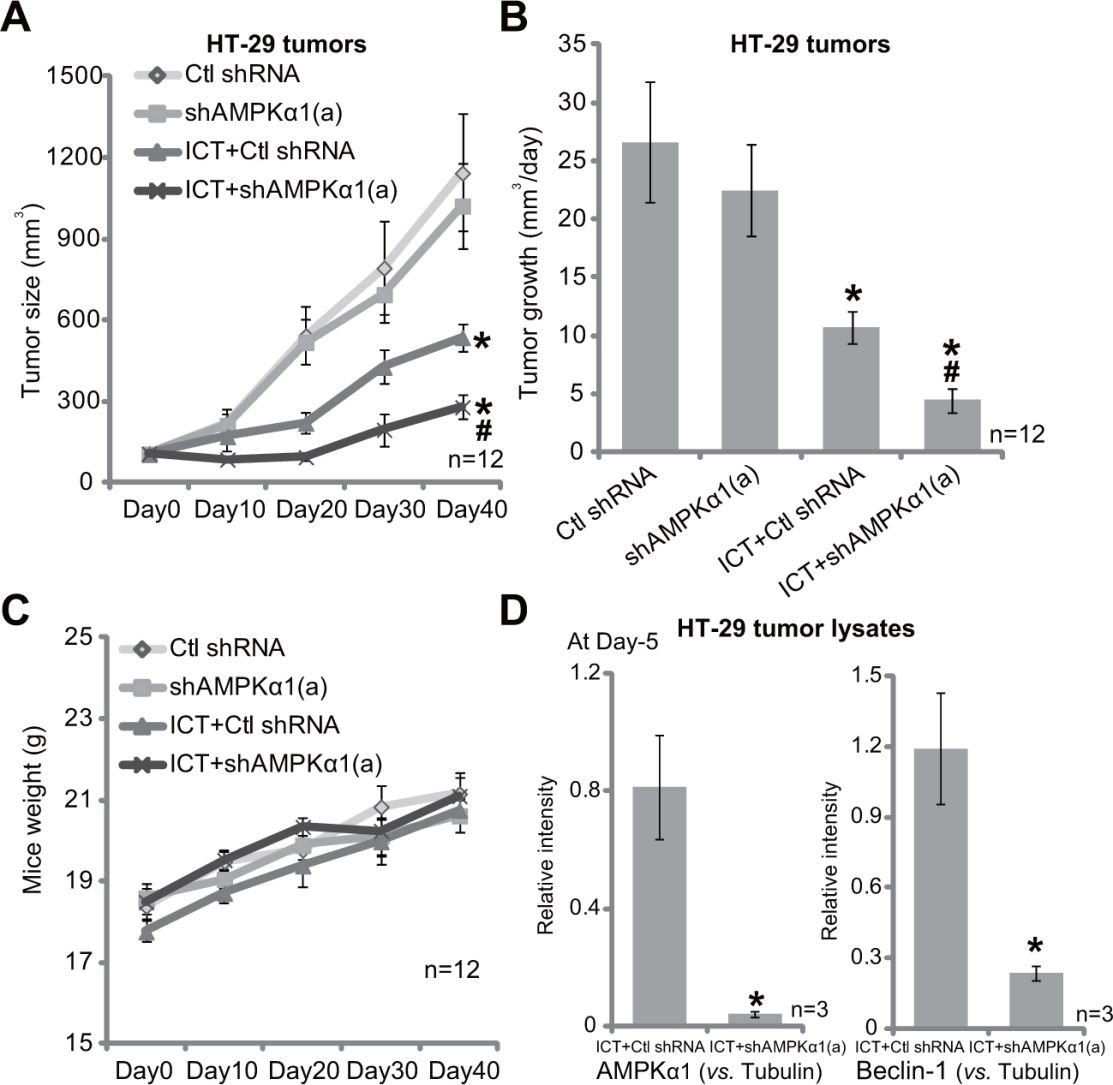
AMPKα1 silence sensitizes icaritin-induced anti-HT-29 tumor activity *in vivo* Nude mice bearing HT-29 tumors, expressing non-sense control shRNA (“Ctl shRNA”) or AMPKα1 shRNA (“a”), were administrated daily with ICT (10 mg/kg, oral gavage) for 21 consecutive days; Tumor volume (**A**) and mice body weight (**C**) were recorded every 10 days for a total of 40 days; Tumor daily growth was also calculated (**B**). Day-5 after initial ICT administration, HT-29 tumors of three mice of ICT treatment group were isolated, expression of listed proteins in fresh tumor lysates was tested, and results were quantified (**D**, three repeats). Data were expressed as mean ± SD. *p < 0.05 vs. “Ctl shRNA” only group. ^#^*p* < 0.05 vs. “ICT” treatment of “Ctl shRNA” group.

Above *in vitro* studies imply that AMPK-autophagy inhibition could sensitize icaritin's anti-CRC cell activity. We next tested this hypothesis *in vivo*, using the HT-29 xenograft model [10]. Tumor growth curve results in Figure 6A demonstrated that oral administration of icaritin (10 mg/kg, daily, for 21 days) in the nude mice inhibited growth of HT-29 xenografts with scramble non-sense control shRNA (“Ctl shRNA”). Remarkably, icaritin-induced anti-tumor activity was dramatically potentiated in HT-29 tumors with AMPKα1 shRNA-a (Figure 6A). Daily tumor growth results further confirmed that AMPKα1 silence sensitized icaritin-mediated anti-HT-29 tumor activity (Figure 6B). Notably, there was no significant difference in the growth of HT-29 tumors expressing AMPKα1 shRNA or Ctl shRNA (Figure 6A and 6B). Intriguingly, the mice body weight was not significantly different among the four groups (Figure 6C), neither did we notice any apparent toxicities. These results imply that these nude mice were well-tolerated to the above treatment regimens. When analyzing tumor tissue lysate samples, we showed that AMPKα1 was indeed silenced in HT-29 tumors expressing AMPKα1 shRNA-a (Figure 6D, quantified data of three repeats). Meanwhile, icaritin-induced autophagy activation, evidenced by strong Beclin-1 expression, was also significantly attenuated with AMPKα1 shRNA knockdown (Figure 6D, quantified data of three repeats). These results indicate that activation of AMPK is possibly also important for autophagy activation and icaritin resistance *in vivo*.

## DISCUSSION

Several inhibitors were applied here. Chloroquine (Cq) and ammonium chloride (NH_4_Cl) both increase intra-lysosomal pH to block degradation of autophagic proteins [36]. On the other hand, 3-methyladenine (3-MA) inhibits LC3B-I to LC3B-II conversion, and inhibits autophagosome initiation [37]. Here, treatment of these autophagy inhibitors dramatically potentiated icaritin-induced lethality against primary and established CRC cells. More intriguingly, pharmacological autophagy inhibition could restore CRC cell apoptosis following icaritin treatment. Therefore, feedback autophagy activation could be a primary reason of non-apoptosis induction in icaritin-treated CRC cells, as reported in our previous study [10]. Inhibition of autophagy then re-provokes apoptosis with icaritin treatment in CRC cells.

In the process of autophagy, autophagosome, the double-membrane structure, will enclose the cellular components [17, 21, 22]. These autophagosomes will then be fused within lysosome to digest the enclosed components, and to supply nutrients for cell survival and apoptosis prevention [17, 21, 22]. Both A*TG-5* and Beclin-1 [27] are indispensable for autophagosome formation [17, 21, 22]. In the current study, we showed that shRNA stable knockdown of ATG-5 or Beclin-1, which presumably blocked autophagy progression, dramatically potentiated icaritin-induced CRC cell death and apoptosis. These genetic evidences further confirm that feedback autophagy activation serves as a primary resistance factor of icaritin in CRC cells.

AMPK is a metabolic switcher that senses change in the intracellular AMP/ATP ratio [38]. Recent studies have proposed a key function of AMPK in the activation of autophagy [31]. There are at least two ways for AMPK to activate autophagy. AMPK phosphorylates and activates its downstream Ulk1, the latter is able to initiate cell autophagy [39, 40]. Secondly, AMPK phosphorylates and in-activates TSC2, which then leads to mTORC1 (mTOR complex 1) inhibition [41, 42]. Activation of mTORC1 will silence autophagy, and AMPK-induced mTORC1 inactivation could then provoke autophagy [39, 40]. Here, we found that icaritin activated AMPK cascade in CRC cells, which served as the upstream signaling for autophagy. Blockage of AMPK activation by AMPKα1 shRNA/siRNA largely inhibited icaritin-induced autophagy activation, yet exacerbating CRC cell death and apoptosis. In nude mice, AMPKα1 shRNA knockdown in HT-29 tumors facilitated icaritin-induced inhibition on tumor growth. Thus, AMPK activation mediates autophagy induction and icaritin resistance in CRC cells. Further studies will be needed to explore the underlying mechanisms of AMPK activation by icaritin, and how activated-AMPK provokes autophagy in icaritin-treated cells.

In summary, our results suggest that feedback activation of AMPK-autophagy cascade could be a major resistance factor of icaritin in CRC cells. Blockage of this signaling dramatically sensitizes icaritin-induced anti-CRC cell activity *in vitro* and *in vivo*.

## MATERIALS AND METHODS

### Chemicals and reagents

Icaritin with a purity of 99.5% was obtained from Shenogen Pharma Co (Beijing, China). icaritin preparation *in vitro* and *in vivo* was described previously [10]. Autophagy inhibitors chloroquine (Cq), ammonium chloride (NH_4_Cl) and 3-methyaldenine (3-MA) were obtained from Sigma-Aldrich (St. Louis, MO). Compound 13 was from Dr. Zhao's group [43]. MHY1485 was purchased from Biotools (Shanghai, China). Antibodies utilized in this study were all obtained from Cell Signaling Tech (Santa Cruz, CA).

### HT-29 cell culture

HT-29 cells were maintained in DMEM medium with 10% FBS, in a CO_2_ incubator.

### Primary colon cancer and epithelial cell culture

Colon cancer tissues from written-informed patients (two patients, Patient-1/−2, both male, 57/48 years old) were washed, and digested as described [24]. Afterwards, individual cells were pelleted and cultured [44]. The primary human colon epithelial cells were provided by Dr. Wei-Hao Sun at the First Affiliated Hospital of Nanjing Medical University (Nanjing, China) [45]. The primary colon epithelial cells were cultured as described [46]. The study was approved by the institutional review board of all authors’ institutions, and was in accordance with the principles expressed in the Declaration of Helsinki. Enrolled patients didn't receive chemotherapy or radiation prior to the surgery.

### Detection of autophagic cells

The light chain 3 (LC3)-GFP-pcDNA3-puromycin, a gift from Dr. Shen [47], was transfected to HT-29 cells or primary colon cancer cells via Lipofectamine 2000 protocol. Afterwards, puromycin (5.0 μg/mL) was added for 6–8 days to select stable cells. Cells were then seeded onto confocal cover-slips. Following the applied treatment, GFP-LC3 accumulation was tested by fluorescence microscopy. Autophagic cells was tested by counting the percentage of cells with intense GFP-LC3 puncta, analyzing at least 200 cells per preparation in five independent experiments [24].

### Cell viability assay

Cell viability was tested by the MTT assay as previously described [10].

### TUNEL staining assay

Following treatment of cells, cell apoptosis was tested by the TUNEL staining assay as described [10]. Percentage of TUNEL positive nuclei was recorded, analyzing at least 200 cells per preparation in five independent experiments.

### LDH assay of cell death

Cell death was tested by lactate dehydrogenase (LDH) release assay, the detailed protocol is described in our previous study [10].

### Western blot assay

Aliquots of 30 μg of lysed proteins were separated by 10% SDS polyacrylamide gel and transferred onto PVDF membranes (Millipore, Bedford, MA). After blocking, the membranes were incubated with specific primary and secondary antibodies. The targeted protein band was visualized via enhanced chemiluminescence (ECL) reagents. The intensity of each blot was quantified using ImageJ software after normalization to the corresponding loading control.

### siRNA and transfection

The AMPKα1 siRNA and the negative control scramble siRNA were purchased from Dharmacon Research Inc. (Lafayette, CO). siRNA transfection (200 nM, 48 hours) was performed using Lipofectamine plus reagents based on the previous protocol [48]. The efficiency of siRNA was determined by Western blots.

### shRNA knockdown and stable cell selection

The two different lentiviral Beclin-1 shRNAs (“-a/−b”) were purchased from Santa Cruz Biotech (sc-29797-V, “Beclin-1 shRNA-a”) and Genepharm (Shanghai, China, “Beclin-1 shRNA-b”), respectively. The two different lentiviral ATG-5 shRNAs were gifts from Dr. Qin Jiang's lab [24]. The three non-overlapping AMPKα1 shRNAs were designed and synthesized by Genepharm. The lentiviral shRNA (20 μL/mL medium) was added directly to the cultured HT-29 cells for 24 hours. Afterwards, cells were cultured in fresh medium for additional 24 hours. Stable cells were selected by puromycin (5.0 μg/mL) for 10–12 days. Control cells were infected with same amount of lentiviral scramble control shRNA (sc-108080-V, Santa Cruz, Beijing, China). Expression of targeted protein was verified by Western blot assay.

### HT-29 xenograft

The *in vivo* experimental protocols were approved by the Institutional Animal Care and Use Committee (IACUC) and Ethics Review Board of authors’ institutions. Male nude mice (7–8 week age, 18–20 g weight) were injected subcutaneously with HT-29 cells (4 × 10^6^ cells/mice) expressing AMPKα1 shRNA or scramble non-sense control shRNA. The treatment was initiated 14 days post tumor cell inoculation, when established tumors were around 100 mm^3^ in volume. Treatment protocol was described. Tumor volumes were measured every 10 days by the modified ellipsoid formula: (π / 6) × *AB*^2^ , and *A* is the longest and *B* is the shortest perpendicular axis of a tumor mass [10].

### Statistical analysis

Data were expressed as mean ± standard deviation (SD). Data were analyzed by one-way ANOVA followed by a Scheffe's f-test by using SPSS 18.0 software (SPSS Inc., Chicago, IL). Significance was chosen as *p* < 0.05. IC-50 was also calculated by SPSS 18.0.
